# Immunohistochemical expression of apolipoprotein B and 4-hydroxynonenal proteins in colorectal carcinoma patients: a retrospective study

**DOI:** 10.3325/cmj.2023.64.29

**Published:** 2023-02

**Authors:** Phei Ying Ng, Siti Norasikin Mohd Nafi, Nur Asyilla Che Jalil, Yee Cheng Kueh, Yeong Yeh Lee, Anani Aila Mat Zin

**Affiliations:** 1 Department of Pathology, Universiti Sains Malaysia, Kubang Kerian, Kelantan, Malaysia; 2 Department of Medicine, Universiti Sains Malaysia, Kubang Kerian, Kelantan, Malaysia; 3 Biostatistics and Research Methodology Unit, Universiti Sains Malaysia, Kubang Kerian, Kelantan, Malaysia; 4 Hospital Universiti Sains Malaysia, Kubang Kerian, Kelantan, Malaysia

## Abstract

**Aim:**

To assess the association of the expression of apolipoprotein B (apoB) and 4-hydroxynonenal (4HNE) with the clinicopathological data of patients with colorectal cancer (CRC).

**Methods:**

We obtained 80 CRC histopathological specimens sent to the Pathology Laboratory of Hospital Universiti Sains Malaysia from 2015 to 2019. Data on demographic factors, body mass index (BMI), and clinicopathological characteristics were also collected. Formalin-fixed paraffin-embedded tissues were stained by using an optimized immunohistochemical protocol.

**Results:**

Patients were mostly older than 50 years, male, Malay, and overweight or obese. A high apoB expression was observed in 87.5% CRC samples (70/80), while a high 4HNE expression was observed in only 17.5% (14/80) of CRCs. The expression of apoB was significantly associated with the sigmoid and rectosigmoid tumor sites (*p* =0.001) and tumor size 3-5 cm (*p* =0.005). 4HNE expression was significantly associated with tumor size 3-5 cm (*p* =0.045). Other variables were not significantly associated with the expression of either marker.

**Conclusion:**

ApoB and 4HNE proteins may play a role in promoting CRC carcinogenesis.

Colorectal carcinoma (CRC) is increasing in prevalence, being currently the second most common cancer in Malaysia and the third most common cancer in the world (1,2). The cancer has considerable morbidity and mortality. The most affected age group are patients older than 50 years, and the disease is frequently diagnosed in advanced stages (3,4). An increasingly recognized CRC risk factor is obesity (5) as it can cause aberrant lipid metabolism by speeding up cancer-cell cycles and replication rates (5,6).

Apolipoprotein B (apoB) and 4-hydroxynonenal (4HNE) are markers related to the oxidation of low-density lipoprotein (LDL). ApoB, predominantly synthesized in the intestines and liver (7,8), is the primary protein component of chylomicrons and LDL, particles that transport cholesterol to the outside of the synthesized organs (6,7,9). It is associated with a poor prognosis in metastatic CRC patients (9) and in stage-III and high-risk stage-II CRC patients undergoing curative surgery (10).

4HNE is a lipid peroxidation marker formed when free radicals react with the lipid membrane of cells under oxidative stress (11). It modifies DNA and binds to proteins to form 4HNE protein adducts, which act as growth-regulating signaling factors and cause inflammation and apoptosis (11,12). 4HNE expression was decreased following apocynin treatment and hyperbaric oxygenation in acute kidney injury (13). However, it was increased in non-alcoholic fatty liver disease (11) and in Barrett's esophagus with specific intestinal metaplasia compared with esophageal cancer (14). It was also higher in well- and moderately-differentiated oropharyngeal cancers, but lower in poorly differentiated and advanced cancers (12).

During LDL oxidation, 4HNE forms protein adducts with the lysine of apoB, thus altering the apoB that binds to LDL (15). A previous study assessing apoB and 4HNE found that modified LDL may promote pre-eclampsia in mothers with diabetes (16). Although the link between apoB and 4HNE has been previously shown, the significance of apoB and 4HNE in cancer development remains to be elucidated. Hence, the current study aimed to determine the differential expression of apoB and 4HNE in human CRC tissues and the association of these markers with demographic factors, body mass index (BMI), and clinicopathological data.

## MATERIAL AND METHODS

### Study design and data extraction

This retrospective study was conducted at Hospital Universiti Sains Malaysia (HUSM), a tertiary referral hospital located in the northeastern region of Peninsular Malaysia. The specimens were obtained from the Laboratory Information System (LIS), a database of histopathological specimens of the Pathology Department of HUSM. The study was approved by the Human Ethics and Research Committee of Universiti Sains Malaysia (USM/JEPeM 19060354).

Of the 98 CRC patients registered in the LIS from 2015 to 2019, 18 were not included as the samples were inadequate for IHC staining. This left 80 specimens in the final sample. Demographic factors, BMI, and clinicopathological characteristics were extracted from the medical records.

### Sample collection and preparation

Formalin-fixed, paraffin-embedded tissue blocks of CRC patients were collected from the Pathology Laboratory. Each of the tissue blocks was sectioned into two-micrometer-thick sections on a poly-L-lysine slide by using a microtome (Leica, Harbourfront Centre, Singapore). The tissue slides were then prepared for IHC staining.

### Immunohistochemistry staining

The IHC staining protocol for apoB and 4HNE was optimized previously (unpublished data). The tissue slides were de-waxed in a hot air oven (Bionics Scientific Technologies, Delhi, India) for 20 minutes and deparaffinized with xylene. Then they were rehydrated with decreasing ethanol concentrations: absolute ethanol (HmbG®, Hamburg, Germany), 95% ethanol, 80% ethanol, 70% ethanol, and 50% ethanol, each for 5 minutes. ApoB and 4HNE antigens were retrieved with citrate and Tris-EDTA buffers (both from Dako, Glostrup, Denmark), respectively. The antigen retrieval was carried out by using a heat-induced process that included incubating slides in a decloaking chamber (Biocare Medical, Pacheco, CA, USA) at 121 psi for 30 seconds. Anti-apoB antibody and anti-4HNE antibody (both from Abcam, Cambridge, UK) (17,18) were incubated at 1:100 concentrations for one hour at room temperature. A negative control was created by omitting the antibody. REAL EnVision Detection System (Dako), which employs horseradish peroxidase-conjugated polymer method, was used to detect and visualize the bound antibodies using 3,3′-diaminobenzidine chromogen. The tissue slides were then counterstained with hematoxylin (Merck, Darmstadt, Germany), dehydrated with an increasing ethanol concentration (50% ethanol to absolute ethanol), cleared with xylene, and mounted by using DPX glue.

### Immunohistochemical scoring

A semi-quantitative IHC scoring protocol was modified from previous studies (6,12). The IHC score, which was calculated only for the tumor area and not for the surrounding normal tissue, was based on two parameters: the proportion of positive tumor cells (0: 0% positive cells; 1: 5-25% positive cells; 2: 26-50% positive cells; and 3: ˃50% positive cells) and the intensity grade (0: no staining; 1: mild staining; 2: moderate staining; and 3: strong staining). The immunoreactive score (IRS) was calculated by multiplying the positive proportion score with the intensity grade. The cut-off point for categorization of the scores into two expression groups was the maximum IRS of 9 divided in half. The expression lower than 4.5 was considered low, whereas the expression higher than 4.5 was considered high. To assess the reproducibility, the IHC scoring was evaluated by two independent pathologists, who were blinded to the clinical data. Disagreements were settled by consensus.

### Statistical analysis

The normality distribution of numerical variables was assessed with a histogram, box-plot, and Shapiro-Wilk test. Numerical variables are presented as mean and standard deviation (SD). Categorical variables are presented as frequency (percentage). The association of markers’ expression with demographic factors, BMI, and clinicopathological characteristics of CRC patients was assessed with a two-sided Fisher’s exact test or a Pearson’s chi-square test. A *P* ˂0.05 was considered statistically significant. The analyses were performed with SPSS, version 26.0 (IBM Corp., Armonk, NY, USA).

## RESULTS

**Figure 1:**
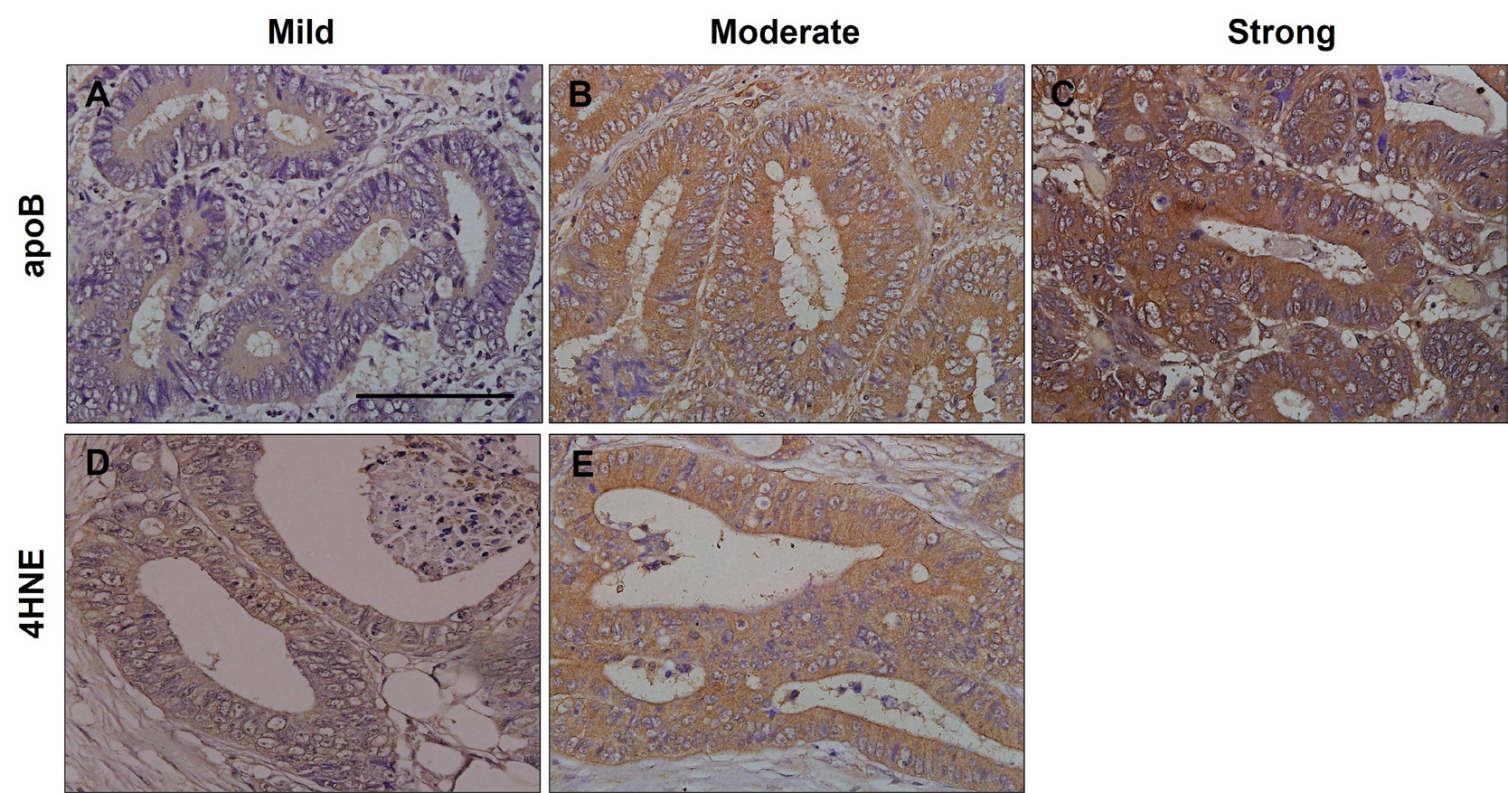
Staining intensities of apolipoprotein B (apoB) (A-C) and 4-hydroxynonenal (4HNE) (D-E) in the cytoplasm of colorectal cancer tissues: (A) mild apoB; (B) moderate apoB; (C) strong apoB; (D) mild 4HNE; and (E) moderate 4HNE (magnification: 40×). Scale bar, 100 μm.

A high expression of apoB was observed in 70 out of 80 CRC samples (87.5%) (Table 1). A high 4HNE expression was observed in only 14 of 80 CRC samples (17.5%) (Table 1). The cytoplasmic staining intensity of apoB was mild, moderate, and strong, while that of 4HNE was mild and moderate (Figure 1).

**Table 1:** Apolipoprotein B (apoB) and 4-hydroxynonenal (4HNE) expression in colorectal carcinoma tissues

Variables	Frequency (n)	Percentage (%)
**ApoB expression**		
**low**	10	12.5
**high**	70	87.5
**4HNE expression**		
**low**	66	82.5
**high**	14	17.5

The majority of CRC patients (76.3%) were older than 50 years at diagnosis, with a mean age of 59 years. The majority were men (53.8%) and members of the Malay subpopulation (90.0%) (Table 2). According to the Asian BMI categorization (19), 56.3% of the CRC patients were overweight or obese (Table 2). However, the expression of apoB or 4HNE was not significantly associated with demographic factors or BMI (Table 2).

**Table 2:** Demographic factors and body mass index (BMI) of colorectal (CRC) patients, and their association with apolipoprotein B (apoB) and 4-hydroxynonenal (4HNE) expression

Variables	CRC patients (n=80), n (%)	ApoB expression, n (%)			4HNE expression, n (%)		
		**low**	**high**	***P***	**low**	**high**	***P***
**Age (mean age ±standard deviation 59±15.03)**				0.999*			0.500*
**≤50**	19 (23.8)	2 (10.5)	17 (89.5)		17 (89.5)	2 (10.5)	
**>50**	61 (76.3)	8 (13.1)	53 (86.9)		49 (80.3)	12 (19.7)	
**Sex**				0.999*			0.779^†^
**male**	43 (53.8)	5 (11.6)	38 (88.4)		35 (81.4)	8 (18.6)	
**female**	37 (46.3)	5 (13.5)	32 (86.5)		31 (83.8)	6 (16.2)	
**Race**				0.065*			0.367*
**Malay**	72 (90.0)	7 (9.7)	65 (90.3)		60 (83.3)	12 (16.7)	
**Chinese**	6 (7.5)	2 (33.3)	4 (66.7)		5 (83.3)	1 (16.7)	
**others**	2 (2.5)	1 (50.0)	1 (50.0)		1 (50.0)	1 (50.0)	
**Asian BMI classification**				0.167*			0.443*
**underweight (˂18.5)**	6 (7.5)	2 (33.3)	4 (66.7)		6 (100.0)	0 (0.0)	
**normal** **(18.5-22.9)**	28 (35.0)	5 (17.9)	23 (82.1)		24 (85.7)	4 (14.3)	
**overweight (23-24.9)**	10 (12.5)	0 (0.0)	10 (100.0)		9 (90.0)	1 (10.0)	
**obese** **(≥25)**	35 (43.8)	3 (8.6)	32 (91.4)		26 (74.3)	9 (25.7)	

*Fisher’s exact test.

†Pearson’s chi square test.

**Figure 2:**
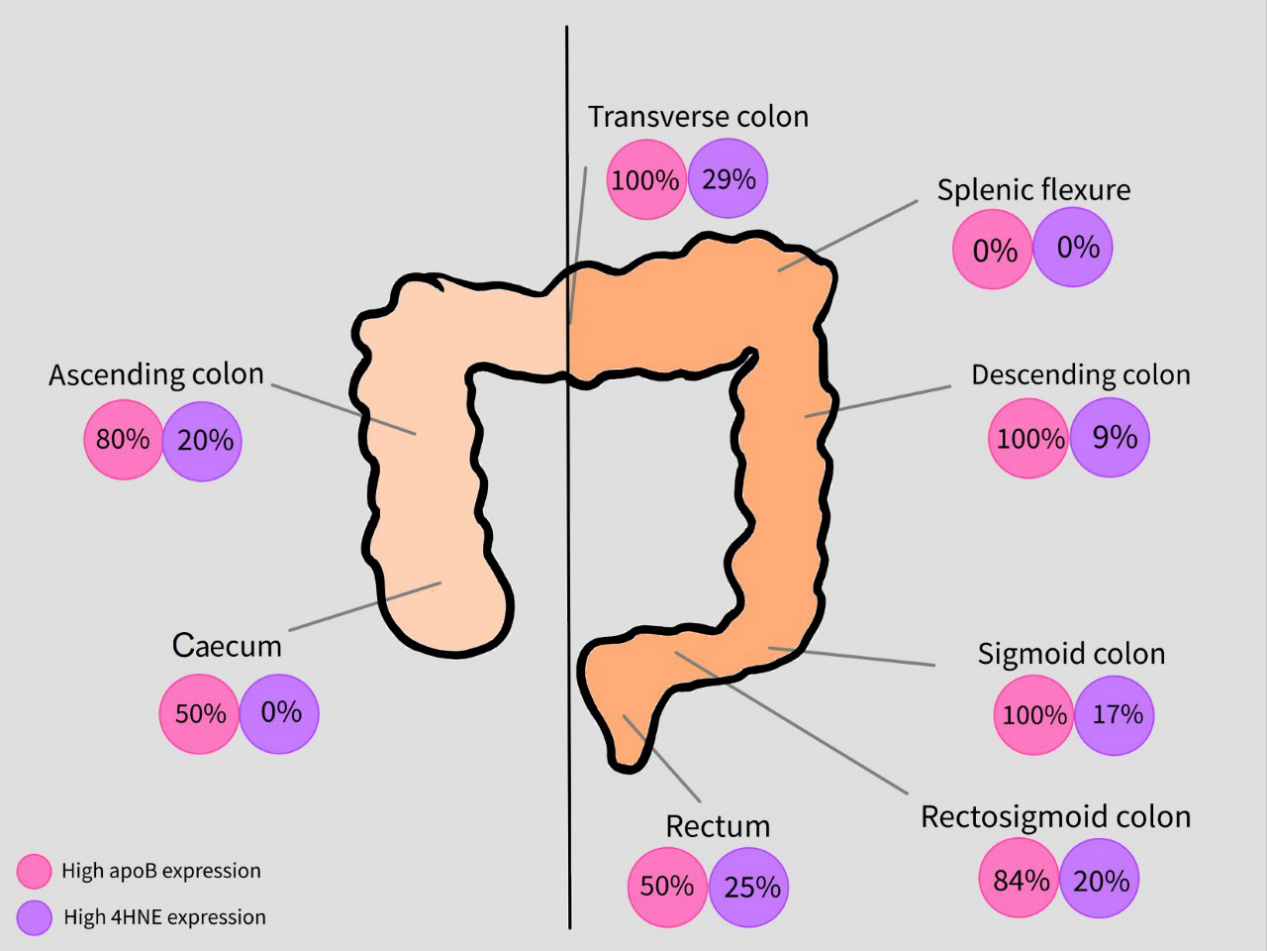
Percentages of high apolipoprotein B (apoB) (pink) or high hydroxynonenal (4HNE) expression (purple) in left-sided and right-sided colorectal cancer (CRC).

The most common sites of CRC were the sigmoid (28.7%) and rectosigmoid (31.3%). High apoB was expressed in all sigmoid tissues and in 84.0% of rectosigmoid tissues. A high apoB expression was observed in most of the left-sided colon, including the sigmoid colon, descending colon, and transverse colon (distal) (Figure 2). The most common CRC tumor sizes were 3-5 cm (47.5%) and >5 cm (48.8%). Overall, 97.4% of CRCs with tumor sizes of 3-5 cm and 82.1% of those with tumor sizes >5 cm demonstrated high apoB expression. On the other hand, 73.7% of CRCs with tumor sizes of 3-5 cm and 92.3% of those with tumor sizes >5 cm expressed low 4HNE expression. ApoB expression was significantly associated with the sigmoid and rectosigmoid tumor site (*p* =0.001) and tumor size of 3-5 cm (*p* =0.005) (Table 3). However, 4HNE expression was significantly associated only with tumor size of 3-5 cm (p =0.045) (Table 3).

**Table 3:** Clinicopathological data of colorectal cancer (CRC) patients and their association with apolipoprotein B (apoB) and 4-hydroxynonenal (4HNE) expression

Variables	CRC patients (n=79)	ApoB expression, n (%)			4HNE expression, n (%)		
		low	high	*P*	low	high	*P*
**Site of tumor**				0.001*			0.937*
**caecum**	4 (5.0)	2 (50.0)	2 (50.0)		4 (100.0)	0 (0.0)	
**ascending colon**	5 (6.3)	1 (20.0)	4 (80.0)		4 (80.0)	1 (20.0)	
**splenic flexure**	1 (1.3)	1 (100.0)	0 (0.0)		1 (100.0)	0 (0.0)	
**transverse colon**	7 (8.8)	0 (0.0)	7 (100.0)		5 (71.4)	2 (28.6)	
**descending colon**	11 (13.8)	0 (0.0)	11 (100.0)		10 (90.9)	1 (9.1)	
**sigmoid colon**	23 (28.7)	0 (0.0)	23 (100.0)		19 (82.6)	4 (17.4)	
**rectosigmoid colon**	25 (31.3)	4 (16.0)	21 (84.0)		20 (80.0)	5 (20.0)	
**rectum**	4 (5.0)	2 (50.0)	2 (50.0)		3 (75.0)	1 (25.0)	
**Size of tumor (cm)**				0.005*			0.045*
**≤ 2**	3 (3.8)	2 (66.7)	1 (33.3)		2 (66.7)	1 (33.3)	
**3-5**	38 (47.5)	1 (2.6)	37 (97.4)		28 (73.7)	10 (26.3)	
**˃5**	39 (48.8)	7 (17.9)	32 (82.1)		36 (92.3)	3 (7.7)	
**Involvement of lymph nodes**				0.310*			0.572**^†^**
**present**	34 (42.5)	6 (17.6)	28 (82.4)		29 (85.3)	5 (14.7)	
**absent**	46 (57.5)	4 (8.7)	42 (91.3)		37 (80.4)	9 (19.6)	
**Invasion of bowel wall**				0.497*			0.580*
**present**	75 (93.8)	9 (12.0)	66 (88.0)		61 (81.3)	14 (18.7)	
**absent**	5 (6.3)	1 (20.0)	4 (80.0)		5 (100.0)	0 (0.0)	
**Lymphovascular invasion**				0.152*			0.381*
**present**	18 (22.5)	1 (5.6)	17 (94.4)		17 (94.4)	1 (5.6)	
**absent**	37 (46.3)	3 (8.1)	34 (91.9)		29 (78.4)	8 (21.6)	
**unavailable**	25 (31.3)	6 (24.0)	19 (76.0)		20 (80.0)	5 (20.0)	
**Perineural invasion **				1.000*			1.000*
**present**	3 (3.8)	0 (0.0)	3 (100.0)		3 (100.0)	0 (0.0)	
**absent**	6 (7.5)	0 (0.0)	6 (100.0)		5 (83.3)	1 (16.7)	
**unavailable**	71 (88.8)	10 (14.1)	61 (85.9)		58 (81.7)	13 (18.3)	
**Tumor types**				0.720*			0.769*
**adenocarcinoma**	71 (88.8)	9 (12.7)	62 (87.3)		57 (80.3)	14 (19.7)	
**mucinous adenocarcinoma**	6 (7.5)	1 (16.7)	5 (83.3)		6 (100.0)	0 (0.0)	
**signet ring cell adenocarcinoma**	2 (2.5)	0 (0.0)	2 (100.0)		2 (100.0)	0 (0.0)	
**mucinous and signet ring cell adenocarcinoma**	1 (1.3)	0 (0.0)	1 (100.0)		1 (100.0)	0 (0.0)	
**Grade of differentiation**				0.724*			0.261*
**well differentiated**	7 (8.8)	0 (0.0)	7 (100.0)		5 (71.4)	2 (28.6)	
**moderately differentiated**	70 (87.5)	10 (14.3)	60 (85.7)		59 (84.3)	11 (15.7)	
**poorly differentiated**	3 (3.8)	0 (0.0)	3 (100.0)		2 (66.7)	1 (33.3)	
**Modified Dukes’ classification**				0.076*			0.681*
**A**	4 (5.0)	1 (25.0)	3 (75.0)		4 (100.0)	0 (0.0)	
**B**	45 (56.3)	3 (6.7)	42 (93.3)		37 (82.2)	8 (17.8)	
**C**	26 (32.5)	4 (15.4)	22 (84.6)		20 (76.9)	6 (23.1)	
**D**	5 (6.3)	2 (40.0)	3 (60.0)		5 (100.0)	0 (0.0)	

*Fisher’s exact test.

†Pearson’s chi square test.

The majority of CRC patients had intestinal wall invasion (93.8%, 75/80) but no lymph node involvement, lymphovascular invasion, or perineural invasion (Table 3). The most common CRC subtypes were adenocarcinomas (88.8%, 71/80); 87.5% (70/80) had moderate differentiation grade and 56.3% (45/80) belonged to modified Dukes’ B class. No other clinicopathological variable was significantly associated with either apoB or 4HNE expression (Table 3).

## DISCUSSION

Our study demonstrated a high apoB IHC expression in the majority of CRC tissues. ApoB expression levels were not significantly associated with age, sex, race, or BMI status. Fang et al (19) also demonstrated no association between serum apoB and demographic factors in CRC patients. However, another study reported significantly higher apoB levels in CRC patients younger than 65 years (20).

A high apoB expression was significantly associated with the sigmoid and rectosigmoid locations, and tumor sizes of 3-5 cm. A high apoB expression was observed in most of the left-sided colon, including the sigmoid colon, descending colon, and transverse colon (distal). In other studies, the right-sided CRC had a poorer prognosis than the left-sided CRC due to a higher rate of metastasis (21,22). Whether high apoB expression in the left-sided CRCs indicates a better prognosis needs to be further studied. However, we also found that tumors with a size of 3-5 cm were more likely to have a high apoB expression than tumors larger than 5 cm, which indicates that the tumor requires apoB at later stages of progression. A lower apoB expression in larger tumors was attributed to lower LDL levels in the late-stage CRC due to poor nutrition (23,24). Later stages of cancer cell growth are presumed to be driven by a *de novo* synthesis of endogenous cholesterol via the 3*-*hydroxy-3-methylglutaryl coenzyme A reductase pathway (23).

4HNE also contributes to the mutagenic and carcinogenic effects of lipid peroxidation (25,26). However, the role of 4HNE in mediating CRC growth is unclear. Increased 4HNE levels were shown to be associated with advanced stages of CRC (27). However, 4HNE was also demonstrated to have an anticancerogenic effect, as shown by its ability to inhibit telomerase activity in intestinal cancer cell lines (26). In KRAS-mutated CRC, 4HNE linked with MAP kinase and transforming growth factor to inhibit tumor growth (28). The involvement of 4HNE in cancer development is also affected by its concentration. At low concentrations, 4HNE has a protective effect and inhibits cancer cell damage, but at high concentrations it can cause apoptosis or necrosis of cancer cells (25). In our study, 4HNE expression was significantly increased in CRC with a size of 2 cm or less. In earlier research, high cytoplasmic 4HNE expression was typically detected in dysplastic and early-stage malignancies, when small tumor sizes are typical (12,29). Due to the toxicity of high 4HNE accumulation, it was hypothesized that 4HNE levels decreased in later stages of cancer progression. The lack of dietary intake of linoleic acid, heme iron, and antioxidants, which may interfere with lipoproteins production, reduced 4HNE production at later stages of CRC (30).

Despite the fact that 4HNE forms protein adducts with apoB to change LDL recognition (15), the role of apoB-4HNE in cancer progression is still unknown. In our study, the intensity of 4HNE protein expression in CRC was lower than that of apoB. Different apoB and 4HNE levels in CRC may indicate that both markers are differently regulated throughout tumor growth. However, the exact mechanism by which 4HNE association with apoB affects cancer growth at the early vs later stages has to be elucidated. In this study, in contrast to apoB, 4HNE expression was not significantly associated with tumor site. This might be explained by previous findings that 4HNE at pathologically relevant concentrations interacts with the cells in the gut. This leads to an elevated inflammatory response and tumorigenesis and results in the progression of inflammatory bowel disease-related CRC (26,30). However, it is not yet understood how changes to the inflammatory response associated with 4HNE in the gut affect CRC.

This study suffers from several limitations. The retrospective design prevented us from collecting sufficient data on certain clinical variables. The small sample size might explain the lack of association of apoB or 4HNE expression with demographic factors, BMI, or clinicopathological features, which was confirmed in other studies. Another limitation was a lack of mechanistic studies to determine the significance of high apoB expression and low 4HNE expression in certain tumor sites and sizes. This research, therefore, could be considered only hypothesis-generating. Although apoB has been linked to CRC development, little is known about its involvement in modulating CRC oncogenic or tumor-suppressor signaling. Attention should be paid to the APOB gene, which is able to impair DNA repair and regulate oncogenic and metastatic regulators (mTOR and PI3K pathways), as well as inhibit tumor suppressors (8,31). As this study only assessed the cytoplasmic expression of 4HNE, future studies should address the co-localization of mitochondrial and cytoplasmic 4HNE by using an immunofluorescence approach. Previous CRC studies found that glycated apoB IHC expression increased from the normal tissue around the cancer site (18%) to the cancer tissue (45%). The same was true for the 4HNE level, as assessed with high-performance liquid chromatography (6,27). We were unable to compare the expression of the antibody markers between normal tissues and colorectal cancer tissues as we focused only on cancer tissues. This issue remains to be investigated in future studies using IHC staining.

In conclusion, apoB expression in the cytoplasm of CRCs was high, but 4HNE protein expression was low, with weaker staining intensity compared with that of apoB. The differences in apoB and 4HNE expressions in different tumor sites and sizes may indicate a role of these proteins in CRC development. Additional research is needed to elucidate the roles of these proteins during colorectal carcinogenesis. Furthermore, apoB and 4HNE expression should be correlated with survival and disease-free times to better comprehend their roles in CRC progression. In addition, since apoB has been associated with the production of lipoproteins in blood, it would be interesting to compare the serum level of apoB and IHC expression of apoB in CRC tissues.
